# Inference of microbial covariation networks using copula models with mixture margins

**DOI:** 10.1093/bioinformatics/btad413

**Published:** 2023-06-28

**Authors:** Rebecca A Deek, Hongzhe Li

**Affiliations:** Department of Biostatistics, Epidemiology and Informatics, Perelman School of Medicine, University of Pennsylvania, Philadelphia, PA 19104, United States; Department of Biostatistics, Epidemiology and Informatics, Perelman School of Medicine, University of Pennsylvania, Philadelphia, PA 19104, United States

## Abstract

**Motivation:**

Quantification of microbial covariations from 16S rRNA and metagenomic sequencing data is difficult due to their sparse nature. In this article, we propose using copula models with mixed zero-beta margins for the estimation of taxon–taxon covariations using data of normalized microbial relative abundances. Copulas allow for separate modeling of the dependence structure from the margins, marginal covariate adjustment, and uncertainty measurement.

**Results:**

Our method shows that a two-stage maximum-likelihood approach provides accurate estimation of model parameters. A corresponding two-stage likelihood ratio test for the dependence parameter is derived and is used for constructing covariation networks. Simulation studies show that the test is valid, robust, and more powerful than tests based upon Pearson’s and rank correlations. Furthermore, we demonstrate that our method can be used to build biologically meaningful microbial networks based on a dataset from the American Gut Project.

**Availability and implementation:**

R package for implementation is available at https://github.com/rebeccadeek/CoMiCoN.

## 1 Introduction

The microbiome, which refers to all the microbiota and their genes in a well-defined environment, has been of great interest in biomedical research (Burge 1988, Lederberg and Mccray 2001). Modern sequencing technologies make it possible to perform large-scale epidemiological microbiome studies, including The Human Microbiome Project and American Gut Project (AGP), both focus on characterizing the microbiome of predominantly healthy subjects (Turnbaugh *et al.* 2007, McDonald *et al.* 2018). Such data provide important information on taxonomic classification and microbial diversity. Differential abundance analysis and the general microbiome association tests have been widely used to understand how the host environment (e.g. human-host health) is associated with the microbiome (White *et al.* 2009, Scealy and Welsh 2011, Paulson *et al.* 2013, McMurdie and Holmes 2014, Peng *et al.* 2016). From such work, it is now known that the human microbiome is associated with complex diseases, such as obesity, inflammatory bowel disease, and rheumatoid arthritis (Scher and Abramson 2011, Greenblum *et al.* 2012, Taneja 2014). Despite these advances much remains unknown about the relationships between microbes and how such relationships change when comparing diseased and healthy microbial communities. What is known is the microorganisms that compose a microbiome form complex and dynamic interactions not only with their host environment but also with one another (Gerber 2014, Li 2015). Such interactions lead to covariations among the bacterial taxa in microbial communities.

Most often, ecological patterns of the microbiome are investigated using microbial covariation, including co-occurrence or co-exclusion. The use of marginal, pairwise association measures are ubiquitous in the analysis of high-throughput sequencing data. Covariation analyses are analogous to co-expression analyses, widely used with gene expression data (Zhang and Horvath 2005). Typically co-occurrence and co-exclusion are defined statistically as a positive or negative correlation, respectively, between two taxa greater than some threshold (Barberán *et al.* 2012, Widder *et al.* 2014, Williams *et al.* 2014). For microbiome studies, the data are often summarized into a vector of bacterial relative abundances, with excessive zeros. There are some attempts to estimate covariations on the absolute abundance scale, using the relative abundance data, by assuming that such covariations are very sparse (Friedman and Alm 2012). These methods are all based on log-ratio transformations (Aitchison 1982, Cao *et al.* 2019). Despite their ubiquity, such transformations are not well suited for data with excessive zeros, as encountered in microbiome relative abundance data. To perform such a transformation, pseudocounts have to be used and their results are difficult to interpret. Even more, the normality assumption of the transformed data often does not hold. Additionally, as shown by Cao *et al.* (2019), the rate of convergence of such a regularized estimate of the covariance matrix with dimension *p* includes the terms $s_{0}(p)\{(\text{log}p)/n\}$ and $s_{0}(p)(s_{0}(p)/p)$, which requires that the sparsity parameter $s_{0}(p)$ is very small as *p* diverges. Such an assumption may not hold for complex microbial covariation networks.

Alternatively, understanding the conditional independence relationship among all the taxa in a microbial community under a given condition may be of interest. Such relationships can be used to address the question of whether the covariation of two taxa can be explained by other taxa (Kurtz *et al.* 2015, Yoon *et al.* 2019). These methods are also based on the log-ratio transformations. Given limited sample sizes and the excessive zeros observed in typical microbiome studies, the multivariate normality assumption does not hold. Further, estimates of such conditional dependence based on regularized estimate of the covariance matrix are very unstable.

Accordingly, due to the reasons outlined above, in this article, we focus on providing a robust and flexible estimate of the covariation of taxon pairs while allowing for covariate adjustments. Instead of attempting to estimate the covariation at the absolute abundance level, which is not possible without strong assumptions, we focus on estimating the covariation at the relative abundance level. Such covariation analyses have appeared widely in microbiome data analysis using relative abundance data and simple correlations (Faust *et al.* 2012, Faust and Raes 2012). Due to excessive zeros in the data, commonly used simple sample correlations may lead to loss of power in identifying covarying taxa pairs. To overcome such limitations, we propose a flexible model-based procedure to estimate the covariation between any two microbes using their normalized relative abundance. Copula models are particularly well suited for this problem as they allow for separate modeling of the univariate marginal distributions from the dependency structure. Unlike existing methods, copulas also allow for covariate adjustment in the margins and uncertainty quantification of their dependence estimate. We perform estimation on the relative abundance scale by modeling the data using a mixture of zeros and a beta distribution, which has been shown to fit microbiome relative abundance data well (Chen and Li 2016, Ho *et al.* 2019). While copulas have been applied to model joint distributions with mixed margins, copula models with both marginal distributions being a mixture of discrete and continuous distributions have not been studied extensively and are the main methodological focus of our paper. We have implemented the methods as an R package with example codes, CoMiCoN (Copulas with Mixture Margins for Covariation Networks), which is available at www.github.com/rebeccadeek/CoMiCoN.

## 2 Copula models with mixture margin distributions

### 2.1 Zero-inflated beta marginal distribution and the copula model

Consider a single microbial sample which can be summarized by the normalized relative abundances of the *m*-microbes, denoted by $(x_{1},\ldots ,x_{m})\in {\left[0,1\right)}^{m}$. We assume that each *x_j_* follows a zero-inflated beta distribution. Accordingly, the marginal density of any given *x_j_* can be written as where we define *p_j_* = Pr$\left(x_{j}=0\right)$, *I_j_* = $I(x_{j}=0)$, and the density function of a beta random variable indexed by mean parameter *μ_j_* and dispersion parameter $\phi_{j}$.


(1) $$f(x_{j})=p_{j}I_{j}+(1-p_{j})f_{\mathrm{beta}}(x_{j};\mu_{j},\phi_{j})(1-I_{j}),$$

$$f_{\mathrm{beta}}(x_{j};\mu_{j},\phi_{j})=\frac{\Gamma (\phi_{j})}{\Gamma (\mu_{j}\phi_{j})\Gamma ((1-\mu_{j})\phi_{j})}x_{j}^{\mu_{j}\phi_{j}-1}{\left(1-x_{j}\right)}^{(1-\mu_{j})\phi_{j}-1}$$

It is often of interest to understand the relationship between any pair of microbes, but calculating the joint distribution of a set of nonnormal random variables can be tedious and contain many parameters. As such, we propose a copula-based approach. Mathematically, a copula is the joint distribution function of a set of uniform random variables, **A** = $\left(a_{i},\ldots ,a_{m}\right)$. Though, in practice, copulas can be used to describe the distribution function of any set of random variables, such that $a_{k}=F_{k}(x_{k})$, where *F_k_* is the marginal cumulative distribution function of the *k*th variable, *x_k_*. This is proven by Sklar’s theorem, which states that any multivariate joint distribution can be described by two parts: (i) the copula function C(·;θ) and (ii) the univariate marginal distribution functions *F_k_* (Sklar 1959). Therefore, for any pair of microbes, we can write the bivariate cumulative distribution of their normalized relative abundances as where $U=F_{i}(\cdot ;\gamma_{i})$ and $V=F_{j}(\cdot ;\gamma_{j})$ are the univariate zero-inflated beta margins of *X_i_* and *X_j_*, respectively, each with parameters $\gamma ={\left(p,\mu ,\phi \right)}^{⊤}$ and C(·;θ) is a family of copula functions with dependence parameter *θ*. The copula function links, or ties, together the margins to form the joint distribution. An advantageous property of copulas is that they completely describe the dependency between the margins via their parameter *θ*, thus allowing for separate modeling of the margins and dependence structure.


$$F(x_{i},x_{j};\gamma_{i},\gamma_{j},\theta_{ij})=C(F_{i}(x_{i};\gamma_{i}),F_{j}(x_{j};\gamma_{j});\theta_{ij})=C(u,v;\theta_{ij}),$$

Moreover, we can specify a set of demographic and clinical variables that affect each microbe’s presence–absence probability, mean abundance, and dispersion using generalized linear models. It is for this reason that we used the alternate parameterization of the beta distribution. We assume that parameters of each margin, *p_k_*, *μ_k_*, and $\phi_{k}$ (k=i,j) can be specified according to a general class of zero-inflated beta regression models as follows (Ospina and Ferrari 2012):


$$h_{1}(p_{k})=f_{1}(Q_{k},\rho_{k});\;h_{2}(\mu_{k})=f_{2}(W_{k},\delta_{k});\;h_{3}(\phi_{k})=f_{3}(Z_{k},\kappa_{k}).$$

We define $Q_{k},\;W_{k}$, and $Z_{k}$ as the matrix of covariates of interest for the presence–absence probability, mean abundance, and dispersion of the *k*th margin, respectively; $\rho_{k},\;\delta_{k}$, and $\kappa_{k}$ as their corresponding vector of regression parameters; and *f*_1_, *f*_2_, and *f*_3_ as some functions of the covariates and regression parameters. As with all GLMs, $h_{1},h_{2}:(0,1)\to R$ and $h_{3}:(0,\infty )\to R$ are strictly monotonic, twice differentiable link functions. Common choices of link function for *h*_1_ and *h*_2_ are the logit, probit, and log–log. Likewise, the log and square-root link functions are common choices for *h*_3_.

### 2.2 Joint density function of bivariate copula model with two mixture marginals

For absolutely continuous margins, the copula distribution function is unique. The joint density function of *X_i_* and *X_j_* can be found by taking mixed partial derivatives of the copula function with respect to *U* and *V*, resulting in $f(x_{i},x_{j})=c(u,v;\theta_{ij})f_{i}(x_{i})f_{j}(x_{j})$, where *c* is the copula density of *C* and *f_i_*, *f_j_* are the marginal densities of *x_i_* and *x_j_*, respectively. For discrete or mixture margins, *C* is not unique and the calculation of the joint density function is not as straightforward.

Gunawan *et al.* (2020) outlines a method for defining the joint density when the margins may belong to any of the three following categories: absolutely continuous, discrete, and mixtures of absolutely continuous and discrete random variables. As such, we can use this general framework to explicitly define the joint density of two zero-inflated beta random variables and use the same notation for consistency. Let M = {i,j} be the index set, C(x) contain the indices of $x=\{x_{i},x_{j}\}$ with continuous *F* at *x*, and D(x) = M−C(x) to be the set of indices of ***x*** for which *F* has a jump point at *x*. Therefore, D is the null set if and only if $x_{i}>0$ and $x_{j}>0$. Using these two sets, Gunawan *et al.* (2020) defines the joint density of *x_i_* and *x_j_* as


(2) $$f(x_{i},x_{j})=c_{C(x)}(b_{C(x)})\prod_{k\in C(x)}f_{k}(x_{k}){△}_{a_{D(x)}}^{b_{D(x)}}C_{D(x)|C(x)}(\cdot |b_{C(x)})$$

Where $a=(F_{i}(x_{i}^{-}),F_{j}(x_{j}^{-}))$ is a vector of cumulative distribution probabilities just before *x_i_* and *x_j_* and $b=(F_{i}(x_{i}),F_{j}(x_{j}))$. Note that when $x_{k}>0,\;F_{k}(x_{k}^{-})=F_{k}(x_{k})$. Moreover, $C_{D(x)|C(x)}$ is the copula conditional distribution function of the point masses at zero conditional on the continuous beta part and ${△}_{a}^{b}g(\cdot )={△}_{a_{i}}^{b_{i}}{△}_{a_{j}}^{b_{j}}g(\cdot )=g(b_{i},b_{j})-g(b_{i},a_{j})-g(a_{i},b_{j})+g(a_{i},a_{j})$. For the bivariate case, this implies that there are four specifications of the joint density (Supplementary data).

The above joint distribution of *x_i_* and *x_j_* holds for any choice of copula function *C*. Although, in this article, we chose to focus on only the Frank copula, whose properties are well suited for microbial covariation. In particular, the Frank copula can model the maximal range of dependence, meaning θ∈{−∞,∞}∖0, with ±∞ corresponding to the Fréchet upper and lower bounds. This is particularly advantageous since other Archimedean copulas, such as the Gumbel and Joe copulas, do not permit negative dependence structures, which are likely to be seen in microbial covariation. Also, the magnitude of dependence is symmetric for positive and negative dependencies, including in the tails of the distribution. We use $C_{Fr}(u,v),\;C_{v|u,Fr}(u,v)$, and $c_{Fr}(u,v)$ to denote the Frank copula distribution function, conditional distribution function, and joint density, respectively, where and $C_{v|u,Fr}(u,v)$ and $c_{Fr}(u,v)$ can be derived.


(3) $$C_{Fr}(u,v)=-\frac{1}{\theta }\text{log}\;\left\{1+\frac{\left(e^{-\theta u}-1)(e^{-\theta v}-1\right)}{e^{-\theta }-1}\right\},$$

Henceforth, we assume that all copulas are referring to the Frank copula. Now that we have defined the bivariate density of *x_i_* and *x_j_*, we can define the likelihood function and use a maximum-likelihood estimation (MLE) procedure for model parameters, $p_{i},\mu_{i},\phi_{i},p_{j},\mu_{j},\phi_{j},$ and *θ_ij_*. In the simplest case, of no covariate adjustment, using the typical full MLE requires a seven-dimensional optimization procedure. The numerical optimization of one function with many parameters is more difficult and computationally intensive than the numerical optimization of several functions with fewer parameters. As such, we use a two-stage, or inference-for-margins, procedure that breaks the parameter estimation into several smaller estimation problems (Shih and Louis 1995, Joe and Xu 1996).

## 3 A two-stage estimation method and statistical inference

### 3.1 A two-stage estimation method

For a sample of size *n*, with observed random vectors $X_{1},\ldots ,X_{n}\in R^{2}$ that represent the relative abundances of a pair of bacteria (*i*, *j*), we consider the univariate log-likelihood functions of the zero-inflated beta margins: and the log-likelihood function for the joint distribution,


$$\ell_{k}(\gamma_{k})=\sum_{l=1}^{n}\;\text{log}\;f_{k}(x_{lk};\gamma_{k}),\;k\in \{i,j\}$$

$$\ell (\theta ,\gamma_{i},\gamma_{j})=\sum_{l=1}^{n}\;\text{log}\;f(X_{l};\gamma_{i},\gamma_{j},\theta ).$$

Note that we have here, and henceforth will, suppress the subscript on *θ*, implying that we are referring to a given (*i*, *j*) pair of microbes, unless otherwise noted. The two-stage estimation procedure (Shih and Louis 1995, Joe and Xu 1996) can be summarized as follows:

1. The log-likelihoods, $\ell_{i}$ and $\ell_{j}$, of the two univariate margins are separately maximized to get estimates of their parameters ${\tilde{\gamma }}_{i}$ and ${\tilde{\gamma }}_{j}$, respectively.
2. The function $\ell (\theta ,{\tilde{\gamma }}_{i},{\tilde{\gamma }}_{j})$ is maximized over *θ* to get $\tilde{\theta }$.

We denote $\eta =(\gamma_{i},\gamma_{j},\theta )$ as the vector of all parameters, $\tilde{\eta }=({\tilde{\gamma }}_{i},{\tilde{\gamma }}_{j},\tilde{\theta })$ as the vector of two-stage estimators, and $\hat{\eta }=({\hat{\gamma }}_{i},{\hat{\gamma }}_{j},\hat{\theta })$ as the MLEs that simultaneously maximize the full log-likelihood function.

We begin with the two-stage MLEs of the *k*th zero-inflated beta margin with log-likelihood $\ell_{k}$ equal to


(4) $$\begin{array}{c} \ell_{k}(\gamma_{k})=z_{k}\;\text{log}(p_{k})+(n-z_{k})\;\text{log}(1-p_{k})+(n-z_{k})\;\text{log}\;\Gamma (\phi_{k})\\ \;-(n-z_{k})\;\text{log}\;\Gamma (\mu_{k}\phi_{k})-(n-z_{k})\;\text{log}\;\Gamma ((1-\mu_{k})\phi_{k})+\\ \;(\mu_{k}\phi_{k}-1)\sum_{l=1}^{n}\;\text{log}\;(x_{lk})+((1-\mu_{k})\phi_{k}-1)\sum_{l=1}^{n}\;\text{log}\;(1-x_{lk}) \end{array}$$

Where $z_{k}=\sum_{l=1}^{n}I_{lk}=\sum_{l=1}^{n}I\;(x_{lk}=0)$ is the number of observations with *x_k_* = 0 and Γ(W+1)=W! is the gamma function. We use the Newton–Raphson algorithm to numerically find the MLEs of $\rho_{k},\delta_{k},$ and $\kappa_{k}$, the GLM regression coefficients.

Now that the marginal two-stage MLEs, ${\tilde{\gamma }}_{k}$, have been defined they can be plugged into the full likelihood $\ell (\theta ,\gamma_{i},\gamma_{j})$ to give:


(5) $$\begin{array}{c} \ell (\theta ,{\tilde{\gamma }}_{i},{\tilde{\gamma }}_{j})\propto \sum_{l\in S1}\;\text{log}\;\{-\theta (e^{-\theta }-1)\}+\sum_{l\in S1}\;\text{log}\;\{e^{-\theta ({\tilde{u}}_{l}+{\tilde{v}}_{l})}\}\\ -\sum_{l\in S1}2\;\text{log}\;\{(e^{-\theta {\tilde{u}}_{l}}-1)(e^{-\theta {\tilde{v}}_{l}}-1)+(e^{-\theta }-1)\}\\ +\sum_{l\in S2}\;\text{log}\;\left\{\frac{e^{-\theta \tilde{p_{i}}}-1}{\left(e^{-\theta \tilde{p_{i}}}-1)(e^{-\theta {\tilde{v}}_{l}}-1)+(e^{-\theta }-1\right)}\right\}\\ +\sum_{l\in S2}\;\text{log}\;\{e^{-\theta {\tilde{v}}_{l}}\}+\sum_{l\in S3}\;\text{log}\;\{e^{-\theta {\tilde{u}}_{l}}\}\\ +\sum_{l\in S3}\;\text{log}\;\left\{\frac{e^{-\theta \tilde{p_{j}}}-1}{\left(e^{-\theta {\tilde{u}}_{l}}-1)(e^{-\theta \tilde{p_{j}}}-1)+(e^{-\theta }-1\right)}\right\}\\ +\sum_{l\in S4}\;\text{log}\;\left\{-\theta \;\text{log}\;\left\{1+\frac{\left(e^{-\theta \tilde{p_{i}}}-1)(e^{-\theta \tilde{p_{j}}}-1\right)}{e^{-\theta }-1}\right\}\right\}. \end{array}$$

The log-likelihood can be split into four parts, each corresponding to the contribution of observations from one of the four possible combinations (Supplementary data). The notation $\sum_{l\in S1}$ implies summation over all the observations that fall into the first scenario, $x_{i}\neq 0$ and $x_{j}\neq 0$, likewise for other summations. We define $\tilde{u_{l}}=F_{i}(x_{li};{\tilde{\gamma }}_{i})$ as the cumulative distribution function of microbe *i* evaluated at *x_li_*, with the two-stage MLEs plugged in for the marginal parameters. The same holds for $\tilde{v_{l}}$ and microbe *j*. Taking the derivative of Equation (5) gives the two-stage score equation for *θ*, which has no closed form solution (Supplementary data). As a result, the two-stage MLE of *θ* is found numerically using a one-dimensional optimizer in R.

### 3.2 Asymptotic normality

Joe (2005) obtained the asymptotic covariance matrix for the vector of two-stage estimators $\tilde{\eta }$ using the theory of inference functions. Specifically, by defining the inference functions where and $g_{\theta }=\partial \;\text{log}\;f(\cdot ;\eta )/\partial \theta$, it is shown that


(6) $$g={\left(g_{i},g_{j},g_{\theta }\right)}^{⊤},$$

(7) $$g_{k}=\frac{\partial \;\text{log}\;f_{k}(\cdot ;\gamma_{k})}{\partial \gamma_{k}},\;\text{for}\;k\in \{i,j\}$$

(8) $$\sqrt{n}(\tilde{\eta }-\eta )\overset{}{\underset{\to }{D}}MVN(0,\Xi ),\;\text{as}\;n\to \infty .$$

Where $\Xi =(-D_{g}{}^{-1})M_{g}{\left(-D_{g}{}^{-1}\right)}^{⊤},\;M_{g}=\text{Cov}(g(Y|\eta ))=E[gg^{⊤}]$, and $D_{g}=E[\partial g(Y,\eta )/\partial \eta^{⊤}]$.

Now let $J=\text{Cov}(g_{i},g_{j})=E[g_{i}g_{j}^{⊤}],\;I=-E[\partial^{2}\;\text{log}\;f/\partial \gamma_{i}\partial \gamma_{j}^{⊤}]$ and $I_{k\theta }=-E[\partial^{2}\;\text{log}\;f/\partial \gamma_{k}\partial \theta ]$ for k=i,j. Then where $a_{k}=-I_{\theta \theta }^{-1}I_{\theta k}J_{kk}^{-1}$ for k=i,j.


(9) $$\begin{array}{c} -D_{g}=\left[\begin{array}{ccc} J_{ii} & 0 & 0\\ 0 & J_{jj} & 0\\ I_{\theta i} & I_{\theta j} & I_{\theta \theta } \end{array}\right],\;-D_{g}{}^{-1}=\left[\begin{array}{ccc} J_{ii}^{-1} & 0 & 0\\ 0 & J_{jj}^{-1} & 0\\ a_{i} & a_{j} & I_{\theta \theta }^{-1} \end{array}\right]\\ \;\;\;\;\;\;\;\;\;\;M_{g}=\left[\begin{array}{ccc} J_{ii} & J_{ij} & 0\\ J_{ji} & J_{jj} & 0\\ 0 & 0 & I_{\theta \theta } \end{array}\right]. \end{array}$$

### 3.3 A rescaled likelihood ratio test

In general, we are interested in determining if any two microbes *i* and *j* have a dependence structure such that $\theta =\Theta_{0}$, for some pre-specified $\Theta_{0}$. We propose a rescaled likelihood ratio test to do so. Consider the general hypothesis testing problem:


$$H_{0}:\theta \in \Theta_{0},\;\text{versus}\;H_{1}:\theta \in \Theta_{1}.$$

Suppose $\ell ={\left(\ell_{i},\ell_{j},\ell_{\theta }\right)}^{⊤}$ where $\ell_{i}$ and $\ell_{j}$ are defined above, and $\ell_{\theta }=\text{log}\;f(\cdot ;\eta )$. Define the two-stage likelihood ratio test statistic as where

Theorem 1. *Under standard regularity conditions, we have* $\Lambda \prime \overset{}{\underset{\to }{D}}\chi_{1}^{2}$.

(10) $$\Lambda \prime =-2\omega [\ell (\theta_{0},{\tilde{\gamma }}_{i},{\tilde{\gamma }}_{j})-\ell (\tilde{\theta },{\tilde{\gamma }}_{i},{\tilde{\gamma }}_{j})],$$

$$\begin{array}{c} \omega =(1+I_{\theta \theta }^{-1}(I_{\theta 1}J_{11}^{-1}I_{1\theta }+I_{\theta 2}J_{22}^{-1}I_{2\theta }+\\ I_{\theta 1}J_{11}^{-1}J_{12}J_{22}^{-1}I_{2\theta }+I_{\theta 2}J_{22}^{-1}J_{21}J_{11}^{-1}I_{1\theta }){)}^{-1}. \end{array}$$

The proof of Theorem 1 can be found in the Supplementary data. It can be shown that the above two-stage likelihood ratio test is equivalent to the pseudolikelihood ratio test (Liang and Self 1996). Most often, the hypothesis we are interested in testing is $\Theta_{0}=\theta_{I}$ where *θ_I_* is the value of the dependence parameter that corresponds to the independence copula. For the Frank copula, this is $\theta_{I}=0$. Under independence, it can be shown that $I_{1\theta }=I_{2\theta }=0$, implying that $\tilde{\theta }$ is asymptotically efficient and the two-stage likelihood ratio statistic reduces to the regular likelihood ratio test statistic (Genest *et al.* 1995, Shih and Louis 1995).

## 4 Simulation studies

Simulation studies were used to assess the performance, in terms of bias and variance, of the two-stage estimation procedure, as well as the type I error and power of the two-stage likelihood ratio test. The data were simulated using the Rosenblatt transformation, a variant of the probability integral transformation (Rosenblatt 1952). Let *U* and *V* be defined as earlier and define a new random variable *W* such that


$$B=C_{v|u}(u,v):=\frac{\partial C(u,v)}{\partial u}=Pr(V=v|U=u).$$

By the Rosenblatt transformation, *U* and *B* are independent uniform random variables and we can define the following simulation algorithm for any two microbes:

1. Simulate U∼ Uniform(0,1) and B∼ Uniform(0,1)
2. Solve for *v* by inverting the conditional copula function such that
  $$v=C_{b|u}^{-1}(b,u)=-\frac{1}{\theta }\text{log}\;\left\{1+\frac{b(e^{-\theta }-1)}{b+e^{-\theta u}(1-b)}\right\}$$
3. Solve for *x_i_* using the definition of *U*:
  $$u=F_{i}(x_{i})\Leftrightarrow x_{i}=F_{i}^{-1}(u)=\{\begin{array}{cc} 0\; & \text{if}\;u\leq p_{i}\\ F_{\text{beta}}^{-1}\left(\frac{u-p_{i}}{1-p_{i}}\right)\; & \text{if}\;u>p_{i} \end{array}$$
  Likewise, the procedure for *x_j_* and *V* is the same.

The process above is repeated for a sample size of *n*. In the event that the simulation scheme above results in <3 nonzero relative abundances for either microbial taxon, the procedure was repeated. This is because at least three nonzero observations are needed to be able to estimate the three taxon-specific marginal parameters. Additionally, for any simulated dataset, if the two taxa are mutually exclusive, meaning no pair of observations have nonzero relative abundance for both taxa, or if only one pair of observations has nonzero relative abundance for both taxa, the procedure was repeated. This was done because such scenarios lead to dependence parameters hitting the lower boundary of estimation and/or cause unstable variance estimates.

Simulations were performed under a variety of marginal parameter settings to understand the robustness of the estimation procedure. The dependence parameter *θ* was selected from {−2.5, −1, 0, 0.5, 1.5, 3}. Under the marginal settings of no covariate adjustment, the zero-inflation probabilities, (*p_i_*, *p_j_*), were selected from {(0.10, 0.25), (0.40, 0.50), (0.60, 0.75), (0.20, 0.75)} and the parameters of the beta portion of the marginal distributions, $(\mu_{k},\phi_{k}),\;k=i,j$, were selected from $\left\{(\frac{2}{7},7),(\frac{5}{7},7),(\frac{1}{2},4),(\frac{1}{3},9),(\frac{2}{3},9),(\frac{1}{2},6)\right\}$.

We also performed simulation with a single continuous covariate affecting the presence–absence probability of each microbe. Under this setting, we assumed that both $Q_{i1}$ and $Q_{j1}$ are drawn from a standard normal distribution, and *p_i_* and *p_j_* were modeled via logistic regressions, $\text{logit}(p_{k}|Q_{k1})=\rho_{k0}+\rho_{k1}Q_{k1},k=i,j$, where $Q_{k1}$ is the confounder variable, $\rho_{k0}$ is the intercept and $\rho_{k1}$ is the coefficient of the confounder. With corresponding vectors of true regression coefficients, $\left\{(\rho_{i0},\rho_{i1}),(\rho_{j0},\rho_{j1})\right\}$, assumed to be from one of the three following settings: {(−0.5,0.7), (−0.3,0.4)},{(−0.1,0.7), (0.1,0.4)}, and {(0.5,0.7), (0.8,0.4)}. In general, these models correspond to low-low, low-high, and high-high zero-inflation probabilities, respectively. The mean abundances were specified as $\mu_{i}=\frac{e^{-0.7}}{1+e^{-0.7}}$ and $\mu_{j}=\frac{e^{-1}}{1+e^{-1}}$ and the dispersion parameters as $\phi_{i}=\phi_{j}=e^{1.5}$. For all parameter settings, the sample size was set to *n *=* *50, except under the setting with no covariates and independence (*θ* = 0) additional simulations were run for a larger sample size of 250. All simulations were repeated 500 times.

### 4.1 Parameter estimation

**Figure 1.**
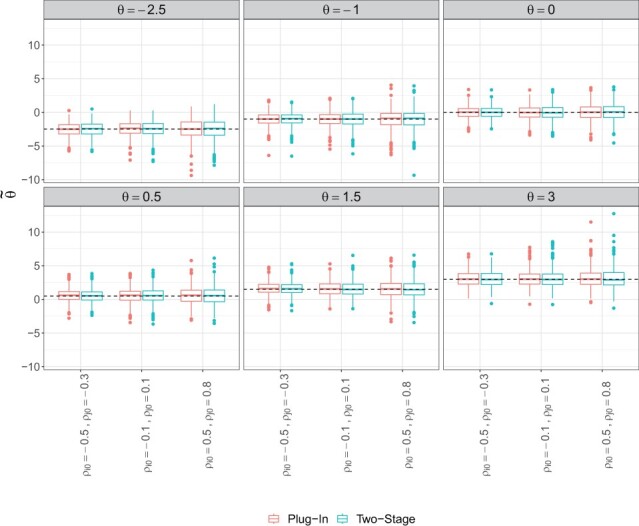
Boxplots of two-stage and plug-in estimated $\tilde{\theta }$ across 500 simulations, where plug-in estimators use the true value of marginal parameters and perform univariate estimation of *θ*. The black dashed line represents the true *θ* value. Data were simulated with covariate adjustment under varying strength of dependence (*θ*) and zero-inflation probability ($\rho_{i0},\rho_{j0}$).

**Figure 2.**
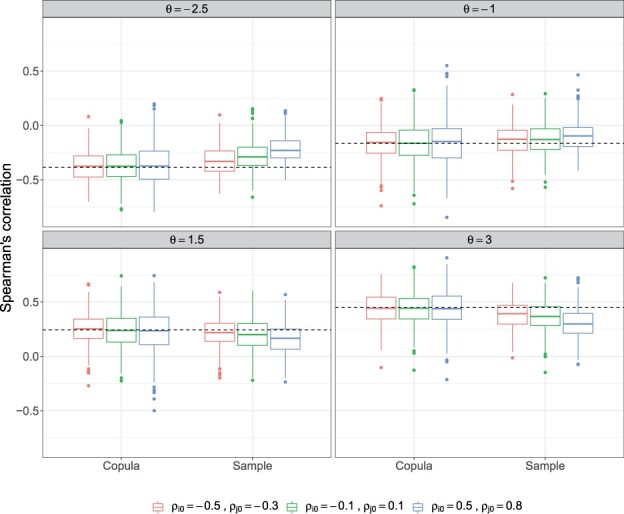
Boxplots of estimated Spearman’s correlation, using copula and sample estimators, across 500 simulations. The black dashed line represents the true value. Data were simulated with covariate adjustment under varying strength of dependence (*θ*) and zero-inflation probability ($\rho_{i0},\rho_{j0}$).

The two-stage estimator is unbiased under all dependence, zero inflation, and marginal parameter settings (Fig. 1 and Supplementary Fig. S1). However, under high zero inflation, we observed some larger outliers in the estimates. This is expected since too many zeros in the data can lead to an unstable estimate of the parameters. The proposed estimator is also performed similar to a plug-in estimator that used the true value of marginal parameters and performs univariate estimation of *θ*. Furthermore, the copula dependence parameter has a relationship to rank correlations such that for a given copula and *θ* value the corresponding Spearman’s rho (*ρ_s_*) and Kendall’s tau (*τ_k_*) can be calculated (Joe 1997). Figure 2 shows for mild to strong dependence structures, the typical sample estimator of Spearman’s correlation is biased, even under low zero-inflation probabilities, while the copula estimator is unbiased. A similar trend holds for Kendall’s tau (results not shown).

In addition to estimating *θ*, we also calculated its variance. The mixed partial derivatives necessary to calculate covariance matrix, Ξ, are analytically difficult to compute, therefore we replace it with a consistent estimator, such as the jackknife estimator:


$$n^{-1}\tilde{\Xi }=\sum_{l=1}^{n}({\tilde{\eta }}^{\left(l\right)}-\tilde{\eta }{)}^{⊤}({\tilde{\eta }}^{\left(l\right)}-\tilde{\eta }).$$

The variance of *θ* is the [7,7] entry of $n^{-1}\tilde{\Xi }$, denoted as ${\hat{\sigma }}_{\theta }^{2}$, and ${\tilde{\eta }}^{\left(l\right)}$ is a vector of two-stage maximum-likelihood estimates calculated with the *l*th observation removed. In general, the variance increased as zero inflation increased, regardless of dependence or marginal parameter values (Supplementary Fig. S2). Specifically, without adjusting for covariates, under high zero inflation of both microbes and moderate-to-strong positive dependence, there was an increase in large outlier estimates (Supplementary Fig. S3). These results show that the mean of the analytical variance is typically larger than the empirical (sample) variance of $\tilde{\theta }$ across all 500 simulations (Supplementary Fig. S4). Though the latter almost always fell within the standard error of the former. The difference between the two increases with zero inflation. This indicates that the jackknife estimator is conservative (upwardly biased) and may lead to a two-stage likelihood ratio test that is conservative as well. As expected, as the sample size increases the variance decreases across the board, though the same trends are seen (results not shown).

### 4.2 Type I error and power

**Figure 3.**
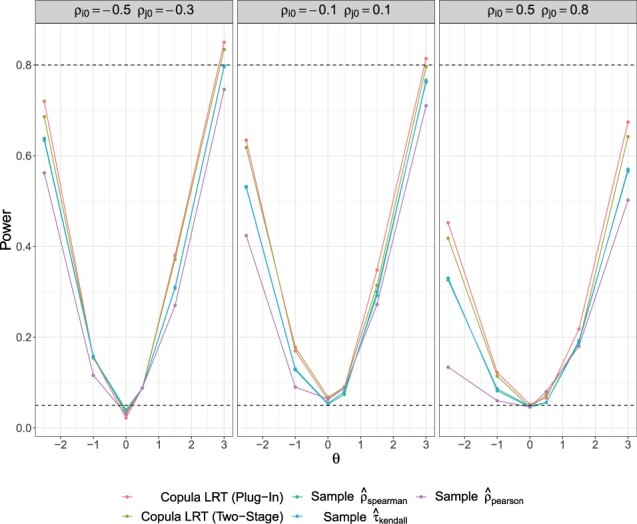
Power curves of the two-stage likelihood ratio test for independence compared to sample correlation tests. Black dashed lines at 0.05 (type I error) and 0.8. Power was calculated under varying strength of dependence (*θ*) and zero-inflation probability ($\rho_{i0},\rho_{j0}$) with covariate adjustment.

We are also interested in assessing the type I error and power of the two-stage likelihood ratio test. Specifically, we would like to test the null hypothesis that two microbes are independent (i.e. $H_{0}:\theta =0$ for the Frank copula) versus the general two-sided alternative hypothesis that the two microbes are not independent (i.e. $H_{1}:\theta \neq 0$). For the setting with covariate adjustment, our proposed likelihood ratio test uniformly outperforms standard sample correlation tests for independence using Pearson’s correlation, as well as Spearman’s and Kendall’s tau rank correlation (Fig. 3). Under low to moderate zero inflation, as the absolute value of the true *θ* moves away from zero, in either direction, the power of the test increases symmetrically. This does not hold under dual high zero inflation where the power to detect a true positive dependence structure increases much more rapidly than that of a true negative dependence structure. This trend does not hold in the setting without covariates (results not shown), under which the four tests perform comparably. This is likely due to the unique mapping between *θ* and Spearman’s and Kendall’s tau rank-based correlations in such settings. Though, there is a slight improvement in our proposed method under dual-high zero inflation, which corresponds to the setting where sample estimators of rank correlations are biased towards the null value of zero.

### 4.3 Model robustness and comparison

We further investigated the robustness of the proposed method in terms of its ability to recover dependency structures under copula model misspecification. First, we simulated data from an underlying bivariate Gaussian copula, instead of the Frank copula that we use in our method. Model parameters were specified in the same way described previously, assuming covariate adjustment. Second, we simulated data under a multivariate Gaussian copula with zero-beta mixture margins. We set the number of margins to 75 and the simulated data were normalized to have unit sum within samples, emulating microbial relative abundance data. More details on parameter specification can be found in the Supplementary data.

**Figure 4.**
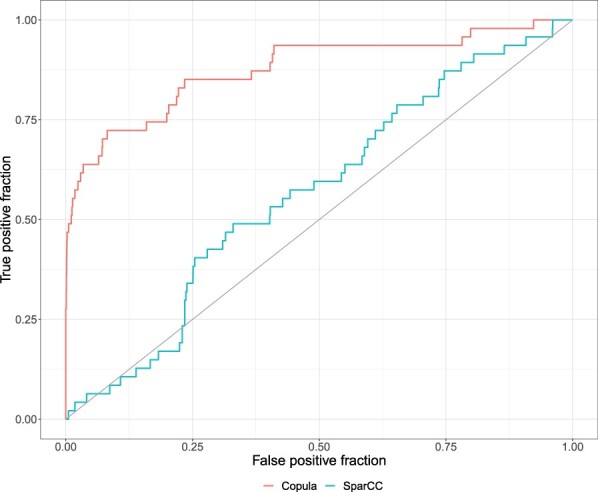
ROC curves based on the two-stage estimator of the copula dependence parameter and SparCC under multivariate Gaussian copula simulation. Cutoffs were selected based on two-stage likelihood ratio test *P*-values for the copula model and the absolute value of the estimated correlation for SparCC, as suggested in the original paper.

We find that even under model misspecification, the proposed method outperforms sample estimators of rank correlations (Supplementary Fig. S5), resulting in less biased estimates of the model parameters and the corresponding Spearman’s correlations. Using multivariate simulation, we investigated the proposed method’s ability to correctly identify significant pairs, across different *P*-value cutoffs. We compare our method with SparCC, as it also focuses on identifying marginal associations using microbial relative abundance data (Kurtz *et al.* 2015). Figure 4 shows the test based on copula model performed well in recovering the pairs with a true dependency (AUC = 0.88), outperforming SparCC which performed only slightly better than random chance (AUC = 0.57). Though, it is important to note that SparCC’s parameter of interest is different than the proposed method’s as it aims to make inference on covariation of the unobserved absolute abundances of the taxa. However, as we discussed in Section 1, SparCC, which depends on center log-ratio transformation of the relative proportions, is only identifiable when the average degree of the correlation network is <1, which is very restrictive.

## 5 Analysis of microbial covariation network in healthy human gut

### 5.1 Identification of pairwise microbial covariation

We used data from the self-selected and open-platform cohort of the AGP to test our proposed method (McDonald *et al.* 2018). The AGP cohort is made up of participants who opted into the study by giving informed consent, as well as paying a fee to cover the cost of sample processing and sequencing. The majority of the samples are from individuals living in the United States, though some samples are from individuals living in the United Kingdom or Australia. The self-reported metadata and 16S rRNA gene sequencing data are accessible from European Bioinformatics Institute under accession number ERP012803.

The data consisted of fecal microbiome samples from 3679 citizen-scientists and 971 unique genera. We filtered the sequencing data such that any reads that were unassigned at the genus level classification were removed. Any genera with a prevalence of <20% across all subjects were removed as well. This left a total of 68 genera for downstream analyses. Furthermore, any samples that had total number of reads of zero after the aforementioned filtering were removed. Since the data also included self-reported metadata, we adjusted for known confounders of the gut microbiome in the marginal regression models. In particular, we adjusted for age (44.6 years ± 17.4), BMI (23.9 ± 5.26), and antibiotic use (69% not in the last year, 14% in the last year, 13% in the last six months, 2% in the last month, and 2% in the last week). Due to the low rate of missing data for each, <5% for age and antibiotic use and about 10% for BMI, we performed a complete case analysis. We further restricted our sample of interest to “healthy” individuals, defined as those who reported not having inflammatory bowel disease or diabetes, as both are known to be associated with dysbiosis. This left 2754 samples remaining.

From these 68 genera, we formed 2278 unique pairs. For each of these pairs, we performed two-stage MLE of the parameters and a likelihood ratio test for independence. Due to the large number of pairwise tests, we adjusted for multiple comparisons by controlling the false discovery rate (FDR) at 1% level. In particular, since the test statistics are not independent from one another we used the Benjamini–Yekutieli procedure (Benjamini and Yekutieli 2001). After FDR control, we identified 1314 pairs of taxa with a significant dependence among healthy subjects.

**Figure 5.**
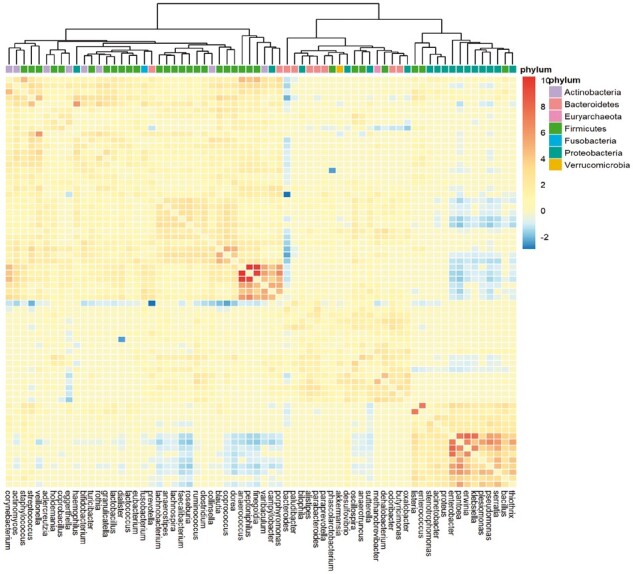
Heatmap of the AGP adjacency matrix with clusters identified by complete agglomerative hierarchical clustering. The side color legend indicates the sign and the magnitude of the estimated dependency parameters for the taxon pairs that are significant, where a value of zero is used for all non-significant pairs.

We compared the results from our method to those from Pearson’s correlation, which detected 276 significant pairs. The two methods have 233 pairs in common, our proposed method identifies 1081 pairs that Pearson’s correlation does not, and Pearson’s correlation identifies 43 pairs not detected by our method. The pairs detected by Pearson’s method, but not by the proposed method have a Pearson’s correlation of between −0.16 and 0.12. The copula estimate of Spearman’s correlation for these pairs are between −0.08 and 0.09, only three of the pairs detected by our method fall in this range. We also compared to SparCC, which detected 86 pairs, all of which were also identified by the copula LRT. Hierarchical clustering of the SparCC adjacency matrix, weighted by the estimated correlation, shows the method misses many of biologically relevant pairs found by the proposed method. The two clusters identified share some similarities to the clusters in Fig. 5, but largely miss pairs between taxa from the same phylum. Lastly, we did not compare to sample rank correlation methods, as simulations showed that in the presence of excessive zeros, their estimation procedure is biased.

### 5.2 Properties of microbial covariation network in healthy human gut

We used the results from the likelihood ratio test for independence to construct a microbial covariation network and adjacency matrix. More specifically, each microbe is a node in the network and two nodes are said to have an edge, or connection, if the result from the microbe pair’s LR test for independence was significant (FDR *P*-value < 0.01). Otherwise, the two nodes are said to be unconnected. A heatmap of the weighted adjacency matrix, where the weight is the estimated *θ* value, shows the covariation relationships between all microbial pairs (Fig. 5). The heatmap was clustered using complete agglomerative hierarchical clustering. Figure 5 shows that the network consists mostly of pairs with positive dependence, especially within clusters, with some negative dependencies between a small set of taxa, mostly between clusters. Furthermore, the microbes (nodes) of the network form three distinct clusters, identified by cutting the dendrogram from hierarchical clustering. The most common phylum in each cluster was *Firmicutes*, *Proteobacteria*, and *Bacteroidetes*. This implies that the clusters have a biological interpretation with taxa of the same phylum tending to be members of the same cluster.

To summarize the resulting network, we calculated the average of node-specific network summary statistics. The network has an average degree of 0.577 (SD = 0.146), average closeness of 0.710 (SD = 0.072), average betweenness of 0.006 (SD = 0.004). The high average degree of the nodes implies the network is dense with many connections. This is further implied by the network’s edge density of 0.58. Meanwhile, the high eigenvalue centrality of 0.704 (SD = 0.198) implies that well connected nodes are likely to be connected with each other. The network also has a diameter of 2 and a mean distance of 1.42.

We simulated 1000 random graphs from the Erdős–Rényi model with the same number of links as the observed AGP network to compare global network measures from these graphs to that of the AGP network. Both the average cluster coefficient (0.695) and modularity (0.137) of the AGP network were significantly different from those of the random graphs (*P *<* *0.001). Thus, implying that the network structure and clusters are not formed due to clustering of random noise in the data. Additionally, we compared the cumulative degree distribution of the AGP network to that of the 1000 random graphs. We observe that the distribution of the random graphs begins ∼35° and increases steeply until it levels off at 50°. In contrast, the distribution of the AGP network begins early ∼20° and rises slowly until a maximum of ∼60°.

### 5.3 Consistency analysis

To assess the robustness and consistency of the identified microbial pairs to slight changes in the observed data, we took 50 bootstrap samples of the relative abundance data, then repeated the estimation and testing analyses, including FDR control at the 0.01 level. If the identified pairs are truly associated with one another, we should see high consistency, or overlap, in the identified pairs between the original data and bootstrap samples. The average number of significant dependent pairs of taxa across all bootstrap samples, rounded to the nearest integer, is 1335. The minimum number of identified pairs is 1274 and the maximum is 1393. The average overlap and Dice coefficients between the pairs identified in the original data and those of each bootstrap sample is 0.940 (SD = 0.010) and 0.930 (SD = 0.006), respectively. This indicates that the identified significant pairs are robust to small changes in the observed data. Furthermore, of the 1314 microbial pairs identified from original data, 875 of these pairs were also identified in all 50 bootstrap samples and 1071 pairs were identified in over 90% of the bootstrap samples. Only 14 were identified in less than half of the bootstrap samples.

## 6 Discussion

In this article, we described a bivariate copula-based density for microbial relative abundance data using zero-inflated beta margins. As such, this allows for a two-stage MLE procedure and corresponding two-stage likelihood ratio test for the copula dependence parameter. Such tests can be used to identify the covarying pairs of bacteria and the corresponding covariation network. Using zero-beta mixture margins provides a flexible way to capture the characteristic excess of zeros in microbiome data, allows for covariate adjustment via the margins, and uncertainty quantification of the estimator of the dependence parameter.

The low bias and high efficiency of the proposed two-stage estimator of the dependence parameter under unknown margins is a valid, and less computationally intensive, alternative to full MLE. We benchmarked the run time of our estimation and testing procedure for the copula dependence parameter under varying sample size and number of microbial features. For a simulated dataset with 45 features and sample size of 100, our two-stage algorithm ran in just under 3 min. The algorithm runs in about 7 min for a sample size of 500 with the same number of features and in a just over eight minutes for the same sample size but with 75 microbial features. Benchmarking was run on a MacBook Pro with a 2.9 GHz 6-Core Intel Core i9 processor using six cores. We extend current work on copula models with mixed margins, as well as work on copula two-stage estimation with our proposed two-stage likelihood ratio test (Shih and Louis 1995, Joe 2005, Gunawan *et al.* 2020). Simulation studies show under the independence hypothesis the test controls type I error and is more powerful than tests based on sample correlation measures.

While this article focuses on the Frank copula, the methods are general and hold for any Archimedean copula. For example, the Gaussian, *t*-, and Clayton copulas can model positive and negative dependence, but assume tail dependence, which Frank does not. For the marginal models of the taxon relative abundance, we use a mixture of zeros and a Beta distribution, which fits the microbiome relative abundance data well, as shown in this and other papers (Chen and Li 2016, Ho *et al.* 2019). However, the copula model framework and the two-stage estimation methods in this paper can be generalized to any other marginal models for zero-inflated proportion or count data, including the models by Liu *et al.* (2019) and various models reviewed in Neelon *et al.* (2016). Additional extensions include modifications to handle longitudinal data to understand the changes in microbial dynamics or to estimate the conserved covariation networks.

The limitation of 16S rRNA or metagenomic sequencing data is that only relative abundance data are available, leading to typical compositional data in microbiome studies. As we discussed in Section 1, although there are attempts in the literature to estimate the covariation at the absolute abundance level, this is almost impossible unless the covariation network is very sparse with only a few dependent pairs. Our experience is that when the number of taxa is large (e.g. over 70 as in our simulations and real data analysis), the false associations purely due to compositionality is quite minimum, unless a few taxa account for most of the abundance. This is usually not the case in our data analysis. Finally, identifying covariation at the relative abundance level itself is biologically interesting, although such a covariation does not always imply a covariation at the absolute abundance level.

## Supplementary Material

btad413_Supplementary_Data

## Data Availability

All sequence data for the American Gut Project can be found in EBI under project PRJEB11419 and Qiita study ID 10317.
