# Supplementary material for: Inference of microbial covariation networks using copula models with mixture margins
Source: Bioinformatics. 2023 Jun 28;39(7):btad413. doi: 10.1093/bioinformatics/btad413 (PMC10336025; doi:10.1093/bioinformatics/btad413)
Supplement: btad413_Supplementary_Data [file btad413_supplementary_data.pdf]

Inference of Microbial Covariation Networks Using Copula Models with Mixture Margins

Supplementary Materials

Rebecca A. Deek and Hongzhe Li

1 Supplementary data

1.1 Copula joint density function

For our specified zero-beta mixture margins, we can define four different joint densities based upon which component(s) of the pair are from the discrete zero portion and differentiation of the copula distribution function. Recall from the manuscript that $\mathcal{M} = \{i, j\}$ is the index set, $\mathcal{C}(\boldsymbol{x})$ contains the indices of $\boldsymbol{x} = \{x_i, x_j\}$ with continuous F at x, and $\mathcal{D}(\boldsymbol{x}) = \mathcal{M} - C(\boldsymbol{x})$ is the set of indices of $\boldsymbol{x}$ for which F has a jump point at x. We now define the four possible joint densities as follows:

- **S1**: $x_i \neq 0, x_j \neq 0, \implies \mathcal{C} = \{i, j\}, \mathcal{D} = \emptyset$

$$f(x_i, x_j) = c(F_i(x_i), F_j(x_j))f_i(x_i)f_j(x_j)$$

- **S2**: $x_i = 0, x_j \neq 0, \implies \mathcal{C} = \{j\}, \mathcal{D} = \{i\}$

$$\begin{aligned} f(x_i, x_j) &= f_j(x_j) \triangle_{F_i(x_i^-)}^{F_i(x_i)} C_{i|j}(\cdot|F_j(x_j)) \\ &= f_j(x_j)\{C_{i|j}(F_i(x_i)|F_j(x_j)) - C_{i|j}(F_i(x_i^-)|F_j(x_j))\} \\ &= f_j(x_j)\{C_{i|j}(p_i|F_j(x_j)) - C_{i|j}(0|F_j(x_j))\} \\ &= f_j(x_j)C_{i|j}(p_i|F_j(x_j)) \end{aligned}$$

- **S3**: $x_i \neq 0, x_j = 0, \implies \mathcal{C} = \{i\}, \mathcal{D} = \{j\}$

$$\begin{aligned} f(x_i, x_j) &= f_i(x_i) \triangle_{F_j(x_j^-)}^{F_j(x_j)} C_{j|i}(\cdot|F_i(x_i)) \\ &= f_i(x_i)\{C_{j|i}(F_j(x_j)|F_i(x_i)) - C_{j|i}(F_j(x_j^-)|F_i(x_i))\} \\ &= f_i(x_i)\{C_{j|i}(p_j|F_i(x_i)) - C_{j|i}(0|F_i(x_i))\} \\ &= f_i(x_i)C_{j|i}(p_j|F_i(x_i)) \end{aligned}$$

- **S4**: $x_i = 0, x_j = 0, \implies \mathcal{C} = \emptyset, \mathcal{D} = \{i, j\}$

$$\begin{aligned} f(x_i, x_j) &= \triangle_{F_i(x_i^-)}^{F_i(x_i)} \triangle_{F_j(x_j^-)}^{F_j(x_j)} C(\cdot) \\ &= \triangle_{F_i(x_i^-)}^{F_i(x_i)} C(\cdot, F_j(x_j)) - C(\cdot, F_j(x_j^-)) \\ &= C(F_i(x_i), F_j(x_j)) - C(F_i(x_i), F_j(x_j^-)) - C(F_i(x_i^-), F_j(x_j)) + C(F_i(x_i^-), F_j(x_j^-)) \\ &= C(p_i, p_j) - C(p_i, 0) - C(0, p_j) + C(0, 0) \\ &= C(p_i, p_j) \end{aligned}$$

1.2 Score equation of the dependence parameter

Score equation with respect to dependence parameter θ is given by:

$$\begin{aligned}\tilde{U}_\theta = &\frac{n_1}{\theta} - \frac{n_1 e^{-\theta}}{e^{-\theta}-1} - \sum_{i\in S1}(\tilde{u}+\tilde{v}) - 2\sum_{i\in S1}\frac{-(\tilde{u}+\tilde{v})e^{-\theta(\tilde{u}+\tilde{v})} + \tilde{u}e^{-\theta\tilde{u}} + \tilde{v}e^{-\theta\tilde{v}} - e^{-\theta}}{e^{-\theta(\tilde{u}+\tilde{v})} - e^{-\theta\tilde{u}} - e^{-\theta\tilde{v}} + e^{-\theta}} \\
&+ \sum_{i\in S2}\frac{-\tilde{p}_i e^{-\theta\tilde{p}_i}}{e^{-\theta\tilde{p}_i}-1} - \sum_{i\in S2}\tilde{v} - \sum_{i\in S2}\frac{-(\tilde{p}_i+\tilde{v})e^{-\theta(\tilde{p}_i+\tilde{v})} + \tilde{p}_i e^{-\theta\tilde{p}_i} + \tilde{v}e^{-\theta\tilde{v}} - e^{-\theta}}{e^{-\theta(\tilde{p}_i+\tilde{v})} - e^{-\theta\tilde{p}_i} - e^{-\theta\tilde{v}} + e^{-\theta}} \\
&+ \sum_{i\in S3}\frac{-\tilde{p}_j e^{-\theta\tilde{p}_j}}{e^{-\theta}-1} - \sum_{i\in S3}\tilde{u} - \sum_{i\in S3}\frac{-(\tilde{u}+\tilde{p}_j)e^{-\theta(\tilde{u}+\tilde{p}_j)} + \tilde{u}e^{-\theta\tilde{u}} + \tilde{p}_j e^{-\theta\tilde{p}_j} - e^{-\theta}}{e^{-\theta(\tilde{u}+\tilde{p}_j)} - e^{-\theta\tilde{u}} - e^{-\theta\tilde{p}_j} + e^{-\theta}} \\
&- \frac{n_4}{\theta} + \sum_{i\in S4}\frac{-(\tilde{p}_i+\tilde{p}_j)e^{-\theta(\tilde{p}_i+\tilde{p}_j)} + \tilde{p}_i e^{-\theta\tilde{p}_i} + \tilde{p}_j e^{-\theta\tilde{p}_j}}{log\left\{1+\frac{e^{-\theta(\tilde{p}_i+\tilde{p}_j)} - e^{-\theta\tilde{p}_i} - e^{-\theta\tilde{p}_j}+1}{e^{-\theta}-1}\right\}\left(1+\frac{e^{-\theta(\tilde{p}_i+\tilde{p}_j)} - e^{-\theta\tilde{p}_i} - e^{-\theta\tilde{p}_j}+1}{e^{-\theta}-1}\right)} \\
&+ \sum_{i\in S4}\frac{e^{-\theta(\tilde{p}_i+\tilde{p}_j)+1} - e^{-\theta(\tilde{p}_i+1)} - e^{-\theta(\tilde{p}_j+1)} + e^{-\theta}}{log\left\{1+\frac{e^{-\theta(\tilde{p}_i+\tilde{p}_j)} - e^{-\theta\tilde{p}_i} - e^{-\theta\tilde{p}_j}+1}{e^{-\theta}-1}\right\}\left(1+\frac{e^{-\theta(\tilde{p}_i+\tilde{p}_j)} - e^{-\theta\tilde{p}_i} - e^{-\theta\tilde{p}_j}+1}{e^{-\theta}-1}\right)(e^{-\theta}-1)^2}\end{aligned}$$

1.3 Proof of Theorem 1

Proof. For simplicity, we write $\ell(\theta) = \ell(\theta, \tilde{\boldsymbol{\gamma}}_i, \tilde{\boldsymbol{\gamma}}_j)$. By Taylor expansion, we have

$$\ell(\theta_0) = \ell(\tilde{\theta}) + (\theta_0 - \tilde{\theta})\ell'(\tilde{\theta}) + \frac{1}{2}(\theta_0 - \tilde{\theta})^2\ell''(\tilde{\theta}) + \dots$$

Since $\tilde{\theta}$ is the value that maximizes $\ell(\theta, \tilde{\boldsymbol{\gamma}}_i, \tilde{\boldsymbol{\gamma}}_j)$, we have $\ell'(\tilde{\theta}) = 0$ and

$$\Lambda = -2[\ell(\theta_0) - \ell(\tilde{\theta})] \asymp -(\theta_0 - \tilde{\theta})^2\ell''(\tilde{\theta}) = -\frac{n(\tilde{\theta}-\theta_0)^2}{\xi}\frac{\ell''(\tilde{\theta})\xi}{n}.$$

where $\xi = \boldsymbol{\Xi}_{7,7}$ is the $(7,7)^{th}$ entry of the covariance matrix of $\tilde{\boldsymbol{\eta}}$, which can be calculated as:

$$\xi = \mathcal{I}_{\theta\theta}^{-1} + \mathcal{I}_{\theta\theta}^{-2}(\mathcal{I}_{\theta 1}\mathcal{J}_{11}^{-1}\mathcal{I}_{1\theta} + \mathcal{I}_{\theta 2}\mathcal{J}_{22}^{-1}\mathcal{I}_{2\theta} + \mathcal{I}_{\theta 1}\mathcal{J}_{11}^{-1}\mathcal{J}_{12}\mathcal{J}_{22}^{-1}\mathcal{I}_{2\theta} + \mathcal{I}_{\theta 2}\mathcal{J}_{22}^{-1}\mathcal{J}_{21}\mathcal{J}_{11}^{-1}\mathcal{I}_{1\theta}). \quad (1.1)$$

Note that $\frac{n(\tilde{\theta}-\theta_0)^2}{v} \xrightarrow{D} \chi_1^2$. Thus, it suffices to deal with the ratio $\frac{-\ell''(\tilde{\theta})\xi}{n}$. Now since

$$-n^{-1}\ell''(\theta) = -n^{-1}\frac{\partial^2\ell(\theta)}{\partial\theta^2} = -\frac{1}{n}\sum_{l=1}^{n}\frac{\partial^2 \log f(\mathbf{X}_l; \tilde{\boldsymbol{\gamma}}_i, \tilde{\boldsymbol{\gamma}}_j, \theta)}{\partial\theta^2},$$

by the Mean Value Theorem and the Law of Large Numbers,

$$-\ell''(\tilde{\theta})/n \xrightarrow{P} -\ell''(\theta)/n \xrightarrow{P} -\mathbb{E}[\ell''(\theta)] = \mathcal{I}_{\theta\theta},$$

which can be approximated by $\tilde{\mathcal{I}}_{\theta\theta}$ using numerical methods and ξ can be estimated by a consistent estimator, $\tilde{\xi}$, such as a jackknife estimate.

We now define the following two-stage LRT statistic:

$$\Lambda' = (\tilde{\xi}\tilde{\mathcal{I}}_{\theta\theta})^{-1}\Lambda = \tilde{\omega}\Lambda \tag{1.2}$$

The above discussion implies

$$\Lambda' \to_D \chi^2_1, \quad \text{as } n \to \infty. \tag{1.3}$$

□

1.4 Simulations studies

1.4.1 Model Robustness and comparison

We performed additional simulations from a Gaussian copula to better understand the sensitivity of the proposed method to choice of copula function. The Gaussian copula was selected as it can capture positive and negative dependence. In the bivariate setting, marginal parameters were set to the same values described in the main text under simulation from a Frank copula. Given that the range of θ depends on choice of copula, we aimed to match strength of dependence, in terms of Spearman's correlation, across copulas rather than magnitude of θ. This was done by utilizing the relationship between a copula dependence parameter and Spearman's correlation. The six θ values specified under simulation from a Frank copula {-2.5, -1, 0, 0.5, 1.5, 3} where converted to their equivalent Spearman's rho and then mapped to the corresponding value of the dependence parameter from a Gaussian copula.

In the multivariate Gaussian setting, the number of features (microbes) was set to 75 to reflect microbial relative abundance data aggregated to the genus level classification. From these 75 microbes, 2775 pairs can be formed. We assumed that few pairs were truly associated with one another; the off-diagonals of the correlation matrix were generated from a Uniform(0.1, 0.55) $\times$ Bernoulli($p = 0.015$). We further assumed that there is one continuous confounder, drawn from a standard Normal distribution, influencing the zero-inflation probabilities of all microbes. The confounder's corresponding regression coefficient was drawn from a Uniform(1,5) distribution. The model intercept was drawn from a Uniform(-1.3,0.5) distribution. Correspondingly, the model intercept for mean abundance and dispersion were drawn from Uniform(-4.5,-1.5) and Uniform(1,1.5) distributions, respectively. The sample size was set as $n = 100$.

2 Supplementary figures

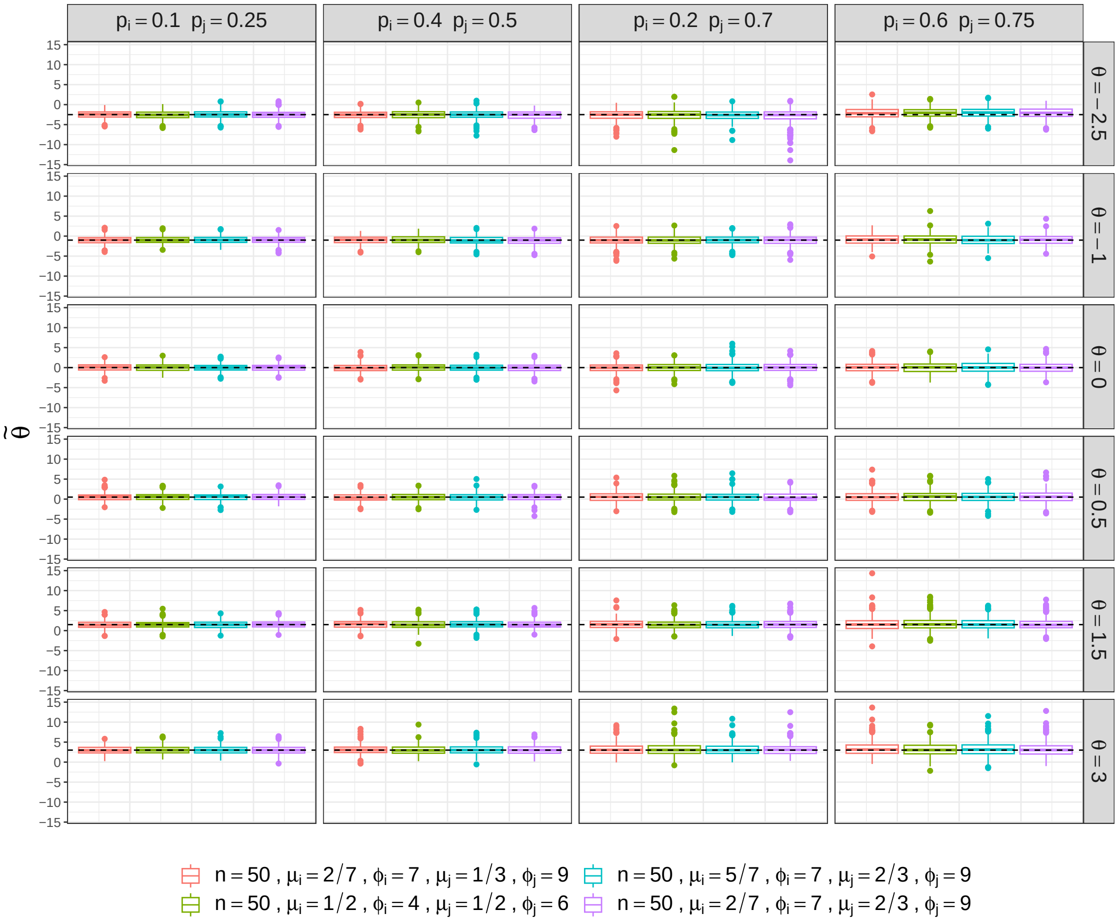

Fig. S1. Boxplot of estimated $\tilde{\theta}$ values across 500 simulations. The black dashed line represents the true θ value. Data was simulated without covariate adjustment under varying strength of dependence (θ), mean (μ), dispersion (ϕ) and zero-inflation probability (p).

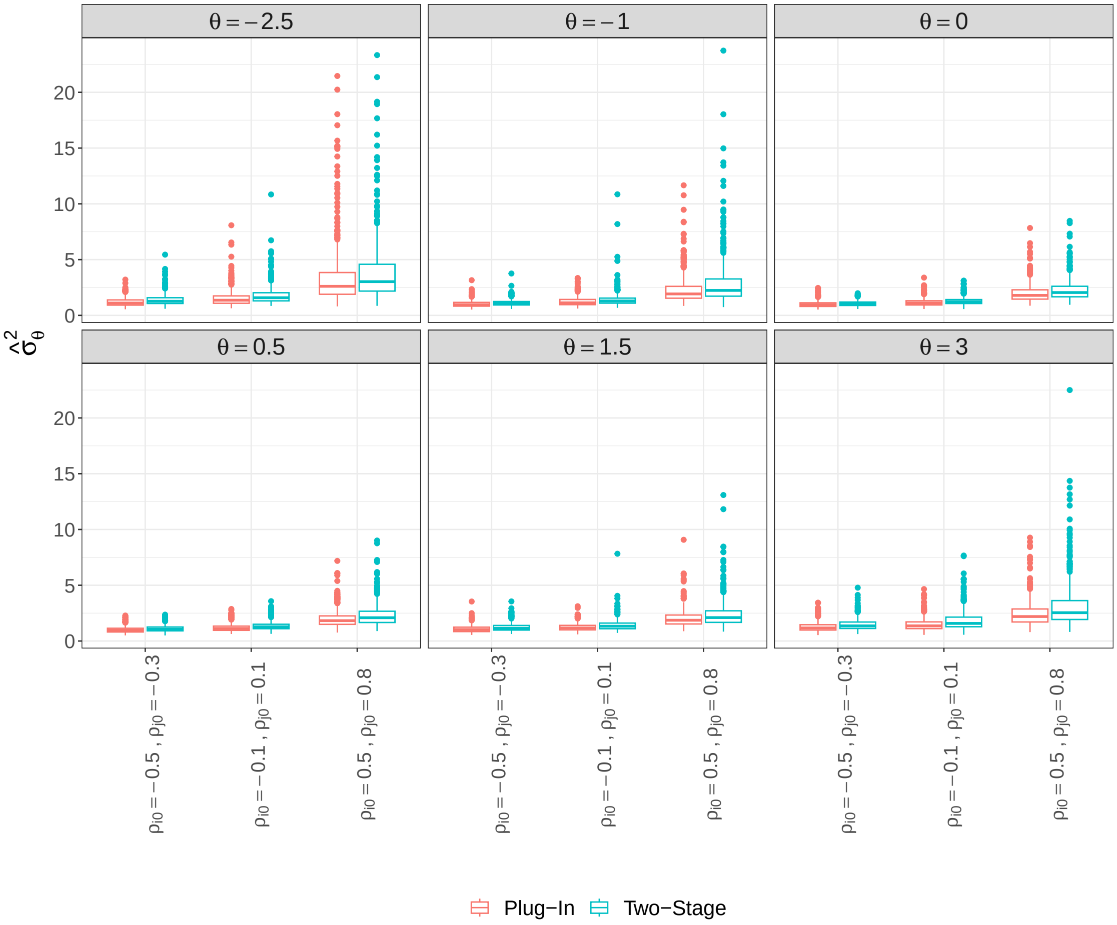

Fig. S2. Boxplots of the jackknife variance of two-stage and plug-in estimated $\tilde{\theta}$, denoted as $\hat{\sigma}^2_\theta$, across 500 simulations. Data was simulated with covariate adjustment under varying strength of dependence (θ) and zero-inflation probability (ρ_{i0}, ρ_{j0}). Outliers with variance values greater than 25 were removed from plots for visualization.

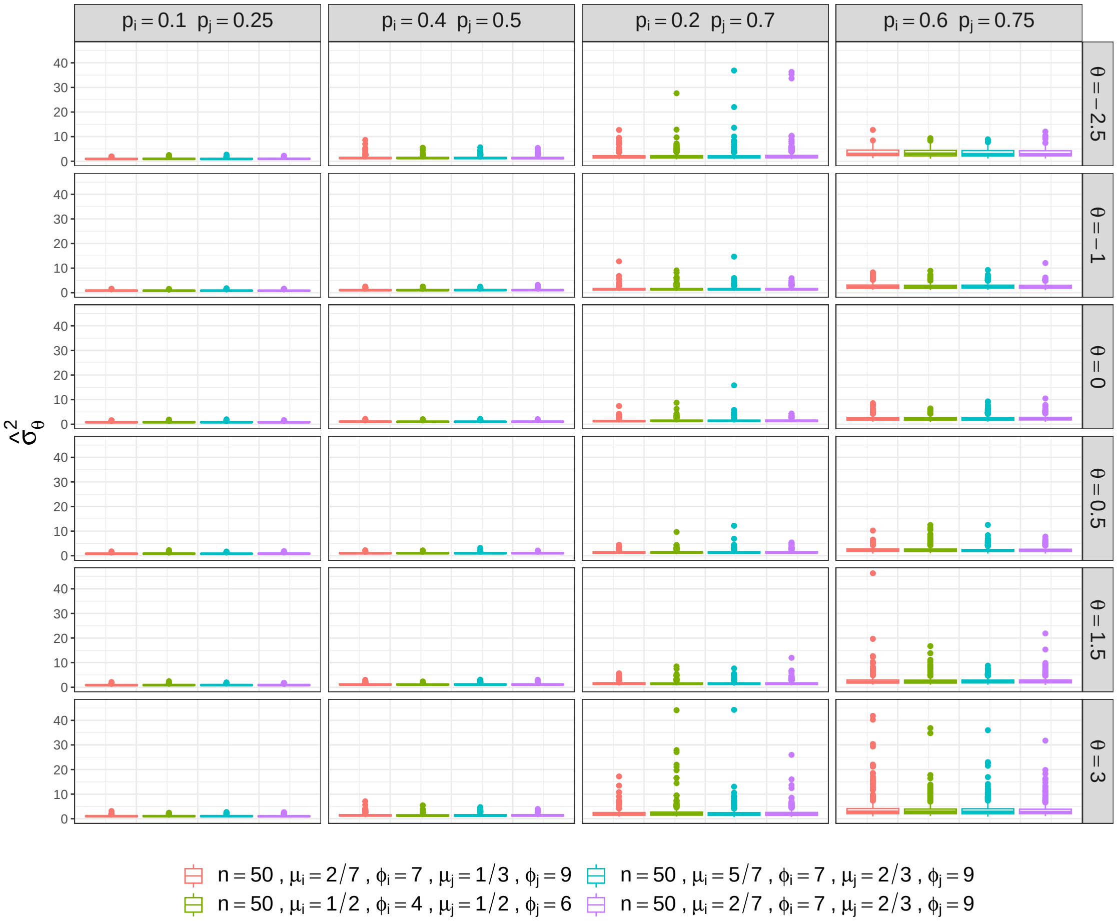

Fig. S3. Boxplot of the estimated jackknife variance of $\tilde{\theta}$, denoted as $\hat{\sigma}^2_\theta$, across 500 simulations. Data was simulated without covariate adjustment under varying strength of dependence (θ), mean (μ), dispersion (ϕ) and zero-inflation probability (p). Outliers with variance values greater than 50 were removed from plots for visualization.

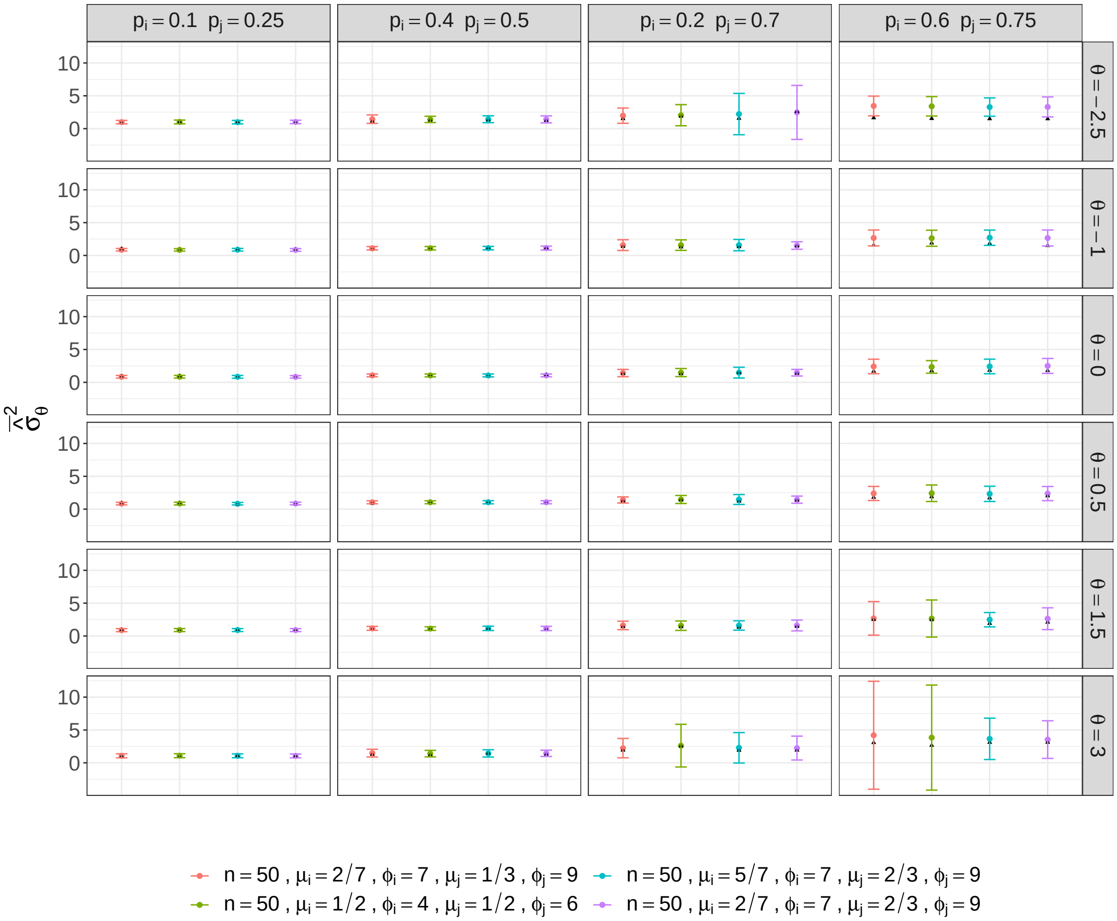

Fig. S4. Mean and standard error bars of the estimated jackknife variance of $\tilde{\theta}$ from data simulated without covariate adjustment under varying strength of dependence (θ), mean (μ), dispersion (ϕ) and zero-inflation probability (p). Black triangles correspond the empirical (sample) variance.

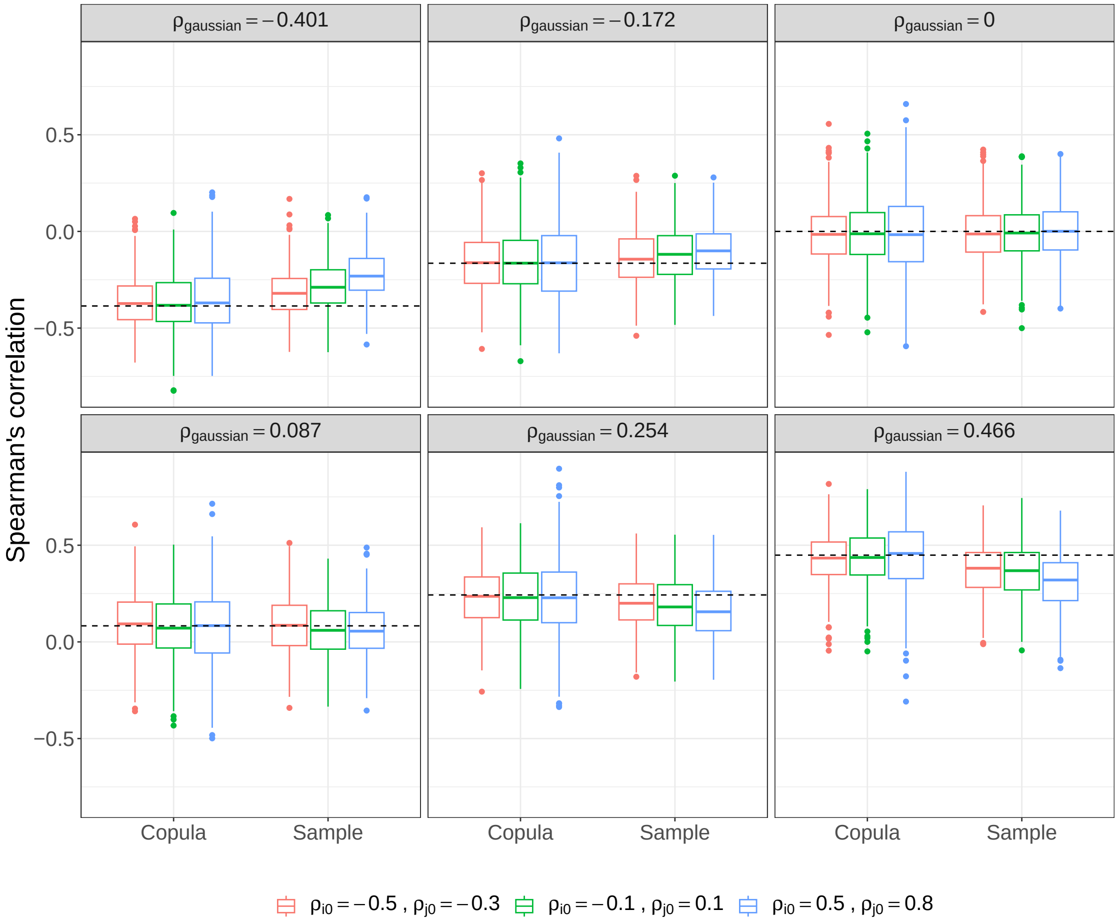

Fig. S5. Boxplot of estimated Spearman's correlation, using copula and sample estimators, across 500 simulations. The black dashed line represents the true value. Data was simulated from a Gaussian copula function with covariate adjustment under varying strength of dependence (θ) and zero-inflation probability (ρ_{i0}, ρ_{j0}).
